# GHSR-Targeted PET Imaging Probe

**DOI:** 10.1021/acsmedchemlett.6c00075

**Published:** 2026-03-16

**Authors:** Ruihu Song, Steven H. Liang

**Affiliations:** † Department of Radiology and Imaging Sciences, 1371 Emory University, Atlanta, Georgia 30322, United States; ‡ Wallace H. Coulter Department of Biomedical Engineering, Georgia Institute of Technology and Emory University, Atlanta, Georgia 30332, United States

**Keywords:** Ghrelin receptor (GHSR), PET, pancreas, diabetes, myocarditis, fluorine 18

## Abstract

Growth hormone secretagogue receptor (GHSR) is expressed in a variety of peripheral organs, the brain, and tumor tissues, making it an important molecular target for both translational imaging and mechanistic studies of GHSR-related physiology and pathology. To date, several GHSR-targeted PET probes have been developed to support disease diagnosis and fundamental research on GHSR function. Recently, a study revealed PET tracer [^18^F]­AQ-12 demonstrated favorable properties, including low lipophilicity, comparable binding affinity to existing ligands, and notably higher pancreatic uptake in GHSR-expressing tissue. Importantly, systematic structure–activity relationship analyses of this compound have been clearly delineated, providing valuable insights to guide further ligand optimization and GHSR-targeted drug development.

## Graphical abstract

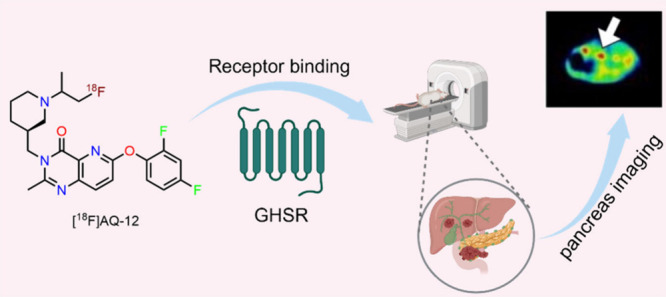

## Introduction

The growth hormone secretagogue receptor (GHSR), a member of the G protein–coupled receptor (GPCR) family, exists in two isoforms: the functional full-length receptor GHSR1a, which contains seven transmembrane domains, and the truncated, nonfunctional variant GHSR1b.^1,2^ GHSR1a is widely distributed in the stomach, intestines, pancreas, heart, lung, brain, and various tumors.^3–5^ The GHSR1a binds to its endogenous ligand, the brain–gut peptide ghrelin, to mediate multiple physiological functions, including stimulation of growth hormone release, regulation of appetite, cardioprotective effects, and modulation of inflammation response (Figure 1).^2,6,7^ GHSR has emerged as a promising target for disease diagnosis and therapy attributed to its widespread tissue distribution and the essential physiological roles of ghrelin. Numerous GHSR-based agonists and antagonists have been developed for treatment of various diseases, such as obesity, epilepsy, diabetes, cardiac disease, and cancer.^4,8–11^ However, the receptor’s physiological functions, pathological alternations, and contributions to disease progression, have not been fully elucidated. This knowledge gap highlights the urgent need for diverse GHSR-targeted probes for noninvasive, real-time, dynamic assessment of receptor expression and function, as well as for the development of GHSR-targeted therapeutics. Positron emission tomography (PET), an advanced molecular imaging technology, offers exceptional sensitivity, quantitative capability, and noninvasive whole body imaging at microdosing levels.^12,13^ The development of GHSR-targeted PET probes represents a valuable approach for enhancing our understanding of ghrelin receptor biology and advancing diagnostic and therapeutic innovation.

**Figure 1:**
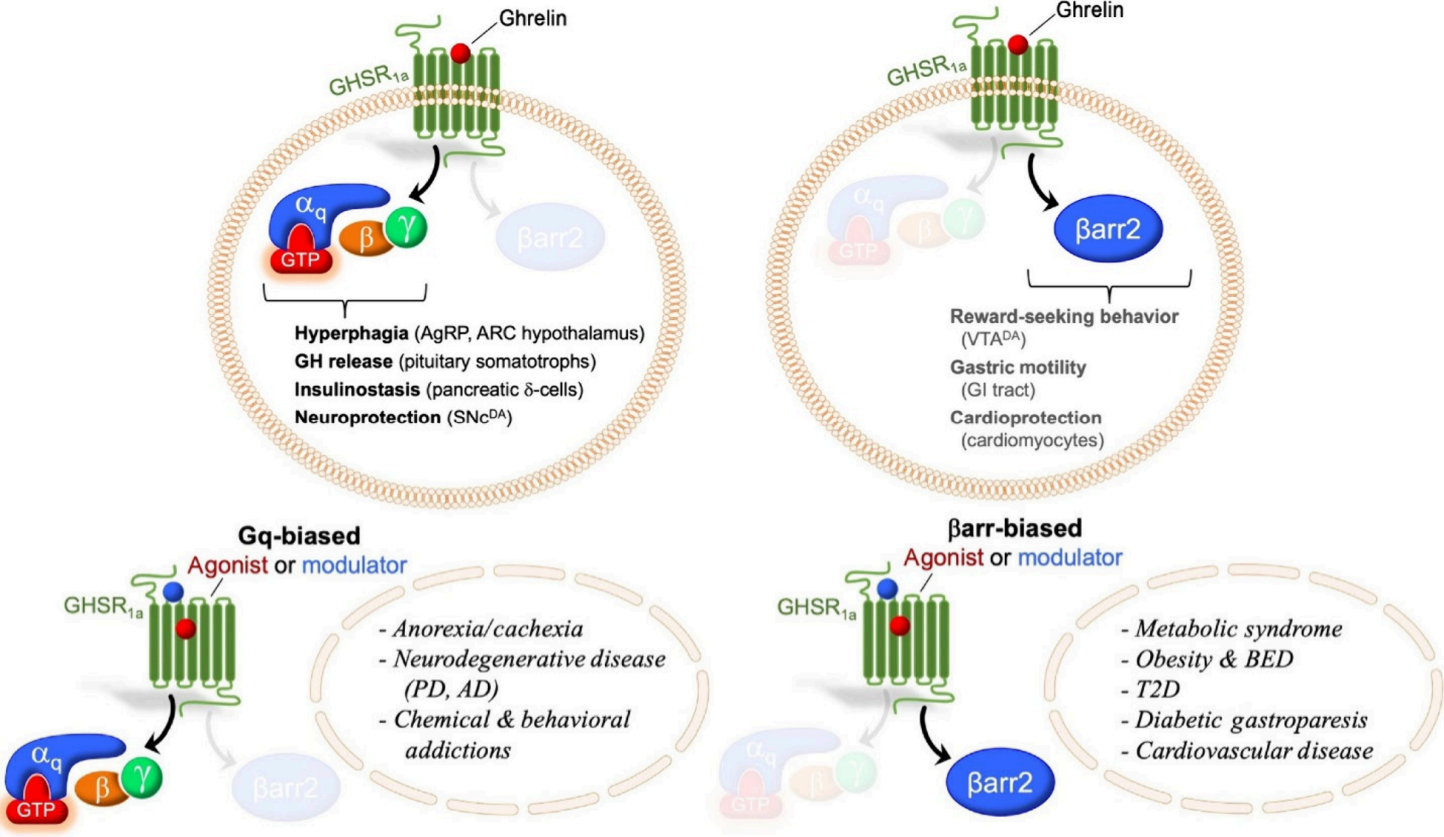
Physiological function and therapeutic applications of biased GHSR1a. Top: the schematic diagram of physiological functions of GHSR and its binding with Ghrelin. Bottom: Agonists and antagonists designed based on their mechanisms for the treatment of related diseases. The figure was adapted from ref 7. Copyright 2022, with permission from Elsevier.

## GHSR-Targeted PET Probe Development

To date, a number of radiolabeled probes targeting GHSR, including ^18^F-labeled compounds such as [^18^F]­1, [^18^F]­LCE470, and [^18^F]­BPP-2, have been developed to investigate the receptor’s physiological functions and involvement diseases (Figure 2A). The probe [^18^F]­1 was first reported in 2017 for imaging GHSR expression in the brain and peripheral organs.^14^ However, a notable accumulation of radioactivity in bone indicated significant *in vivo* defluorination of [^18^F]­1, reflecting limited metabolic stability. Its high cLogP value (6.30) further prevented applications in neuroimaging. To address the issue, another ^18^F-labeled GHSR probe [^18^F]­BPP-2 was designed based on diaminopyrimidine scaffold to enhance hydrophilicity while maintaining high binding affinity for GHSR.^15^ *In vitro* binding studies indicated that the *K*_i_ value of BPP-2 (274 nM) was comparable to that of known ligand Abb8a (108 nM). Molecular docking revealed noncovalent interactions with the GHSR binding pocket, confirming that the fluoroethoxy group did not compromise affinity. However, *in vivo* PET imaging demonstrated limited brain uptake (0.53% ID/g at 30 min postinjection). Peripheral imaging showed moderate pancreas uptake (4.83% ID/g at 10 min postinjection), consistent with GHSR expression. Similar to [^18^F]­1, [^18^F]­BPP-2 also underwent partial defluorination, evidenced by bone radioactivity, highlighting the need for further structural optimization to enhance stability.

**Figure 2:**
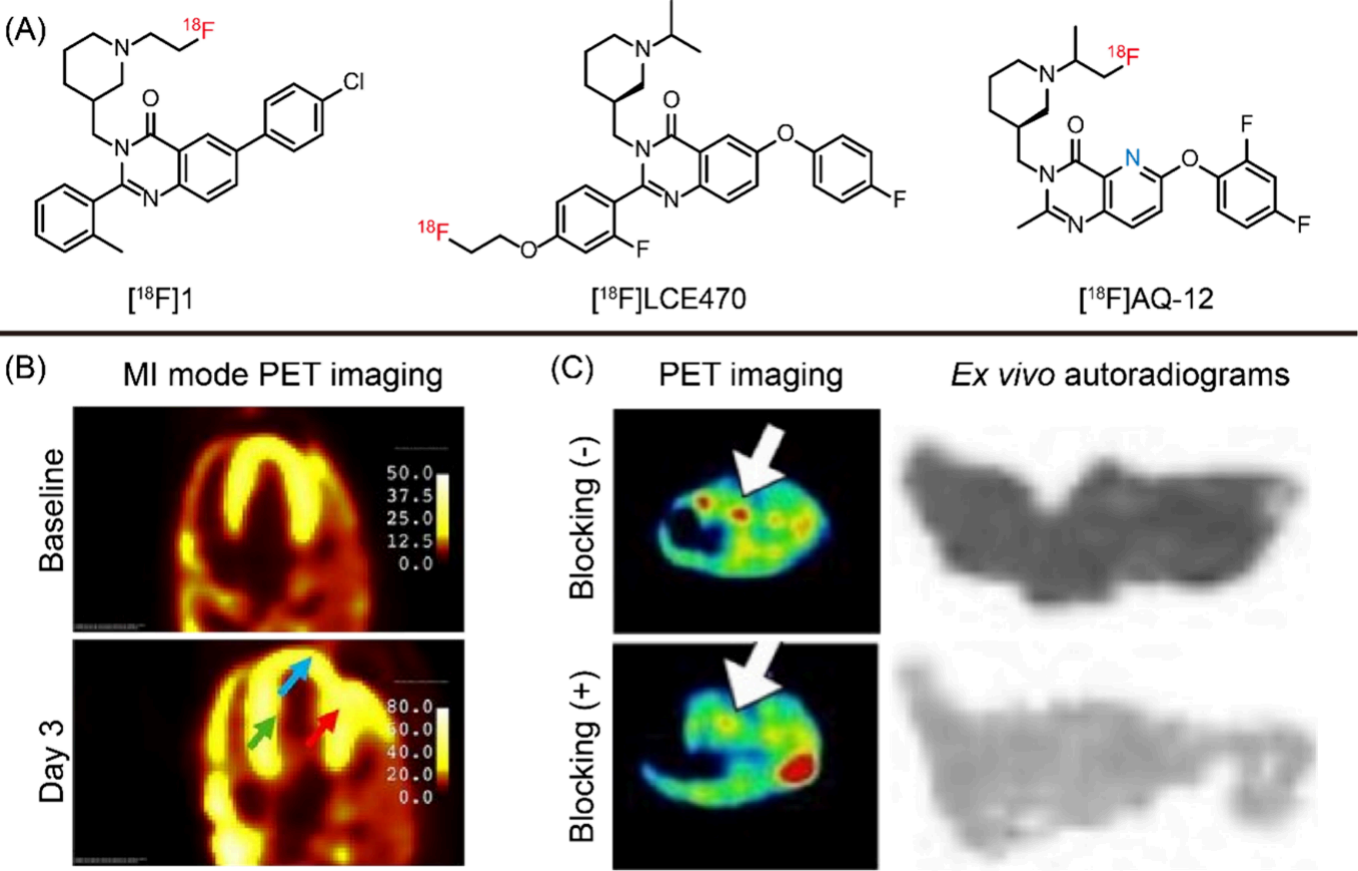
(A) The structure of [^18^F]­1, [^18^F]­LCE470, and [^18^F]­AQ-12. (B) *In vivo* myocardial infarction imaging of [^18^F]­LCE470. (C) *In vivo* PET/CT imaging and *ex vivo* autoradiograms of [^18^F]­AQ-12. Panels B, and C were reproduced with permission from ref 16 (Copyright 2024, the Society of Nuclear Medicine and Molecular Imaging), and^17^ (Copyright 2025, American Chemical Society).

Given ghrelin’s essential role in cardiomyocyte physiology, multiple GHSR-targeted small molecules and peptides have been developed for treating cardiac conditions such as myocarditis and fibrosis. Therefore, GHSR-targeted PET probes serve as valuable tools for assessing therapeutic response and characterizing receptor dynamics in cardiovascular pathology. Building on quinazolinone derivatives originally developed for obesity treatment, several GHSR-targeted compounds were synthesized, among which LCE470 displayed the highest activity (IC_50_ = 0.33 nM) for GHSR and was developed as a PET tracer.^16^ Before myocardial infarction surgery, [^18^F]­LCE470 distributed uniformly across the infarcted region, remote myocardium, and left circumflex coronary artery (LCX), with similar dynamic changes (Figure 2B). Postsurgery, however, the infarct zone displayed a progressive washout pattern, indicating loss of cardiomyocyte function. Quantitative data revealed lower tracer uptake in the infarct area relative to the LCX throughout imaging. Complementary fluorescence imaging studies with Cy5-conjugated ghrelin further demonstrated higher signal in LCX compared to the infarct region, and fluorescence intensity strongly correlated with radioactivity, confirming the high affinity and specificity of [^18^F]­LCE470 for GHSR.

## Literature Highlight: [^18^F]AQ-12

In previous studies, several GHSR-targeted PET probes, including [^18^F]­1 and [^18^F]­LCE470, exhibited high binding affinity and promising imaging performance, yet both suffered from notable nonspecific binding due to the intrinsic high lipophilicity of their structure. The probe [^18^F]­BPP-2 with lower lipophilicity showed reduced nonspecific accumulation but at the cost of weaker binding affinity and *in vivo* defluorination. Building on these earlier probes, new efforts have focused on systematically optimizing probe structure and elucidating structure–activity relationships (SAR) to enhance our understanding of GHSR biology and support rational design of next-generation GHSR-targeted diagnostics and therapeutics. Guided by these principles, a series of azaquinazolinone derivatives were rationally designed and synthesized through structural modifications at multiple positions based on YIL-718, yielding two main types: 5-azaquinazolinone and 5,8-azaquinazolinone derivatives.^17^ Binding assays showed that 5-azaquinazolinone derivatives exhibited higher affinity (K_i_ of AQ-1, AQ-2, and AQ-3 = 33.9, 9.42, and 715 nM, respectively) than 5,8-azaquinazolinone derivatives (AQ-5, AQ-6, and AQ-7: K_i_ = 57.2, 12.8, and 4950 nM, respectively). SAR analysis revealed that introducing an *m*-difluorobenzene substituent at the 6-position of the 5-azaquinazolinone scaffold produced superior binding affinity compared with pyridine, fluorobenzene, or fluoropyridine analogs. Measurements of oil–water partition coefficient further demonstrated that replacing the benzene ring with a pyridine ring, as in AQ-3 and AQ-6, lowered lipophilicity relative to AQ-1 and AQ-5, establishing a close correlation between cLogP values and nitrogen content in the molecular structure. Reduced cLogP values are advantageous because they generally predict lower nonspecific binding to off-target tissues. Among the initial set, AQ-2 emerged as the most promising candidate owing to its excellent affinity and favorable physicochemical properties. Subsequent structural optimization based on AQ-2 resulted in six new 5-azaquinazolinone derivatives and binding assays identified the probes AQ-12, AQ-13, and AQ-14 (K_i_ = 4.73, 8.07, and 6.89 nM, respectively) as top performers, exhibiting higher binding affinities than reported compound 1 (K_i_ = 17.0 nM). These candidates were subsequently radiolabeled with ^18^F, and their stability and specificity were evaluated through *in vitro* and *in vivo* studies. Biodistribution experiments showed that [^18^F]­AQ-12 and [^18^F]­AQ-13 exhibited high pancreatic uptake (23.6 and 9.5% ID/g at 10 min postinjection, respectively), consistent with known high GHSR expression in the pancreas. Meanwhile, the heart and spleen, both organs with moderate GHSR expression levels, exhibited appreciable probe accumulation. In contrast, [^18^F]­AQ-14 showed negligible GHSR-dependent uptake in peripheral organs. These differences in uptake and kinetic profiles among the three probes likely reflect variations in hydrophilicity, ^18^F-labeling position and different substituent groups. Given its superior affinity and favorable *in vivo* profile, [^18^F]­AQ-12 was advanced for further evaluation. PET imaging demonstrated clear visualization of the visualization of the pancreas within 10–15 min postinjection, with blocking studies revealing 57% reduction in pancreatic accumulation at 30 min, confirming target-specific binding (Figure 2C). This imaging performance exceeded that of [^18^F]­LCE470, demonstrating the advantage of [^18^F]­AQ-12. Consistent with PET results, *ex vivo* autoradiography showed homogeneous pancreatic radioactivity in the baseline group and a marked reduction following blocking. These data highlight the excellent specific binding and promising translational potential of [^18^F]­AQ-12 for imaging GHSR in the pancreas and for studying GHSR-related diseases.

## Outlook

GHSR-targeted PET probes demonstrate the promising clinical potential for disease diagnosis and therapeutic response assessment in obesity, myocardial infarction, pancreatic disorders and neurodegenerative diseases, as well as for monitoring dynamic changes in ghrelin signaling. The development of AQ-12 represents a significant advance in GHSR-targeted ligand design; its low lipophilicity, high binding affinity, and high target specificity position it as a strong candidate for PET imaging applications. The clinical translation potential of AQ-12 is further supported by comprehensive biodistribution studies, *in vitro* autoradiography, and *in vivo* PET imaging. The majority of GHSR-targeted PET probes reported to date rely on the quinazolinone scaffold, highlighting the need to expand chemical scaffold diversity to support drug development and enable exploration of new imaging modalities and to accelerate clinical translation and broaden the biological and translational applications of GHSR-targeted PET probes.

## Abbreviations

GHSR: growth hormone secretagogue receptor
PET: Positron emission tomography.
